# Toripalimab plus gemcitabine and cisplatin induction chemotherapy in locoregionally advanced nasopharyngeal carcinoma: a retrospective study

**DOI:** 10.3389/fonc.2025.1704442

**Published:** 2025-11-24

**Authors:** Renba Liang, Ling Lei, Xinxiao Li, Fengming Lan, Fangmeng Fu, Teng Zou, Li Ma, Peng Chen, Zhanmei Wang, Jing Jin, Jianghu Zhang

**Affiliations:** 1 Department of Radiation Oncology, National Cancer Center/National Clinical Research Center for Cancer/Cancer Hospital & Shenzhen Hospital, Chinese Academy of Medical Sciences and Peking Union Medical College, Shenzhen, China; 2 Department of Radiation Oncology, Shenzhen Luohu People’s Hospital, Shenzhen, China

**Keywords:** toripalimab, induction chemotherapy, nasopharyngeal carcinoma, immunotherapy, gemcitabine and cisplatin

## Abstract

**Objective:**

To explore the efficacy and safety of toripalimab combined with gemcitabine and cisplatin (GP) induction chemotherapy and sequential concurrent chemoradiotherapy in LANPC treatment.

**Methods:**

This was a retrospective analysis of 105 patients with LANPC from December 2019 to December 2022. In total, 50 patients received two or three cycles of GP induction chemotherapy and 55 patients received toripalimab plus GP. Toripalimab (240 mg) was given intravenously on the first day of each cycle of induction chemotherapy. All patients received radiotherapy or concurrent chemoradiotherapy with cisplatin.

**Results:**

After induction therapy, 17 (30.9%) patients in the GP plus toripalimab group and 6 (12.0%) in the GP alone group achieved complete response (CR) (p=0.019). After a median follow-up of 38.6 months, 16.0% (8/50) of the patients in the GP group and 3.6% (2/55) of the patients in the toripalimab plus GP group experienced recurrence or metastasis. There were 2 deaths in the GP group and no deaths in the toripalimab plus GP group. The 2-year event-free survival (EFS) rate was higher in the toripalimab plus GP group than in the GP group (98.1% vs. 85.4% (HR, 0.28; 95% confidence interval [CI], 0.08–0.97; p=0.024)). The 2-year overall survival, locoregional relapse-free survival and distant metastasis-free survival rates for toripalimab plus GP vs. GP alone were 100.0% vs. 100.0% (p=1.00), 98.1% vs. 89.5% (p=0.086), and 100.0% vs. 95.9% (p=0.15), respectively. Grade 3–4 adverse events (AEs) occurred in 26 (47.3%) and 29 (58.0%) patients in the toripalimab plus GP and GP alone arms, respectively. The most common grade 3–4 AEs were neutropenia (20 [36.4%] vs. 21 [42.0%]), leukopenia (18 [32.7%] vs. 17 [34.0%]), and vomiting (15 [27.3%] vs. 12 [24.0%]) in the toripalimab plus GP arm compared with the GP alone arm. Immune-related AEs of grade 3–4 occurred in three (5.5%) patients in the toripalimab plus GP arm.

**Conclusions:**

The addition of toripalimab to GP induction chemotherapy significantly improves EFS without increasing toxicity in LANPC.

## Introduction

Nasopharyngeal carcinoma is a malignant tumor that occurs in the nasopharyngeal epithelium. The disease has a special regional distribution and is common in South China, Southeast Asia, and North Africa (1). More than 70% of patients have locoregionally advanced disease at the time of diagnosis.

Nasopharyngeal carcinoma is highly sensitive to ionizing radiation. Radiotherapy or the combination of intensity-modulated radiotherapy (IMRT) and platinum-based chemotherapy is the backbone of treatment for NPC (2, 3). However, for locally advanced NPC (LANPC), distant metastasis and local recurrence are the main failure patterns of therapy (2, 4, 5). Identifying a way to address this issue is a hot topic in clinical research. Studies have shown that the addition of induction chemotherapy (IC) to chemoradiotherapy significantly improves the recurrence-free survival and overall survival of patients with LANPC (6–9).

In recent years, immune checkpoint inhibitors have attracted great interest among researchers. Previous studies, including CAPTAIN-1st, JUPITER-02 and RATIONALE-309, have shown that immune checkpoint inhibitors improve the survival of patients with recurrent or metastatic nasopharyngeal carcinoma (10–15). Nevertheless, in the context of LANPC, the efficacy and safety of gemcitabine and cisplatin induction chemotherapy plus toripalimab are unclear. Hence, we conducted this retrospective study to evaluate the efficacy and safety of adding toripalimab to gemcitabine and cisplatin induction chemotherapy in patients with LANPC.

## Materials and methods

### Patients

This was a retrospective study of the clinical data of patients with locally advanced NPC who received induction therapy at the National Cancer Center/National Clinical Research Center for Cancer/Cancer Hospital & Shenzhen Hospital between December 2019 and December 2022. The inclusion criteria for this study were as follows: (i) age ≥18 years; (ii) pathologically diagnosed with NPC; (iii) stage III/IVa in accordance with the 8th Edition of the AJCC; (iv) Karnofsky performance score (KPS) ≥70; (v) receiving IC or IC plus toripalimab followed by definitive CCRT; (vi) receiving concurrent chemotherapy with cisplatin; and (vii) receiving IMRT. The exclusion criteria were as follows: (i) received concurrent immunotherapy; (ii) received adjuvant chemotherapy or adjuvant immunotherapy after CCRT; (iii) underwent surgery before IC; or (iv) had a second malignancy.

### Treatment

All patients received radical IMRT at our hospital as previously described (16). The radiation used in radiotherapy is 6MV-X rays. Briefly, the radiation doses used were as follows: 69.96 Gy at 2.12 Gy/fraction to the planning target volume (PTV) of the nasopharyngeal gross tumor volume (GTV), 69.96 Gy to the PTV of the GTV of the metastatic lymph nodes, 60.06 Gy to the PTV of the high-risk clinical target volume, and 54.45 Gy to the PTV of the low-risk clinical target volume. If the patient received concurrent nimotuzumab therapy, nimotuzumab (200 mg/week) was given intravenously on the first day of radiotherapy.

The induction chemotherapy regimens used were gemcitabine (1000 mg/m^2^ on days 1 and 8) and cisplatin (80 mg/m^2^ on day 1) every 3 weeks for 2–3 cycles. Concurrent chemotherapy consisted of 100 mg of cisplatin per square meter. Toripalimab (240 mg) was given intravenously on the first day of each cycle of IC.

### Clinical endpoints

The endpoints included event-free survival (EFS, the time from the start of treatment to disease progression or death from any cause), overall survival (OS, the time from the start of treatment to death from any cause), distant metastasis-free survival (DMFS, the time from the start of treatment to distant metastasis) and locoregional recurrence-free survival (LRFS, the time from the start of treatment to locoregional recurrence). Efficacy was evaluated according to the Response Evaluation Criteria in Solid Tumors version 1.1 (RECISTv1.1). Acute toxicities during treatment were graded according to the Common Terminology Criteria for Adverse Events (version 4.0), and late toxicities related to radiotherapy were evaluated on the basis of the Late Radiation Morbidity Scoring Scheme of the Radiation Therapy Oncology Group.

### Statistical analysis

The chi-squared test or Fisher’s Freeman–Halton test was used for the comparison of categorical variables. One-way analysis of variance (ANOVA) was used for the comparison of differences in the numerical variables among groups. Two-group comparisons of the survival data via Kaplan–Meier curves and analyzed by mean of log-rank tests. A multivariate Cox proportional hazards model was used to calculate hazard ratios (HRs), 95% confidence intervals (CIs) and independent prognostic factors. p<0.05 was considered statistically significant.

## Results

### Patient characteristics

In total, 105 patients, including 50 (47.6%) patients with GP alone and 55 (52.4%) patients with GP combined with toripalimab, were eligible for this study. The characteristics of the patients at baseline are summarized in **Table 1**. Most patients had N2 or N3 disease of the cervical lymph nodes or bulky primary tumors (T3 or T4). According to the 8th edition of the AJCC, 31 (29.5%) patients were in stage III, and 64 (61.0%) were in stage IVA. There were no significant differences in age, sex, T stage, N stage, TNM stage, pathological characteristics, cycles of IC, cycles of concurrent chemotherapy, or concurrent nimotuzumab between the two groups.

**Table 1:** Basic characteristics of patients.

Characteristics	To+GP (N = 55) (N, %)	GP (N = 50) (N, %)	P-value
Gender			
Male	39 (70.9)	36 (72.0)	0.90
Female	16 (29.1)	14 (28.0)	
Age, years (Median, Range)	44 (24-73)	45 (33-76)	0.44
Histology (nonkeratinizing)			
differentiated	3 (5.5)	2 (4.0)	0.73
undifferentiated	52 (94.5)	48 (96.0)	
T stage			
T1	9 (16.4)	8 (16.0)	0.73
T2	4 (7.3)	7 (14.0)	
T3	28 (50.9)	23 (46.0)	
T4	14 (25.4)	12 (24.0)	
N stage			
N1	8 (14.5)	7 (14.0)	0.83
N2	21 (38.2)	22 (44.0)	
N3	26 (47.3)	21 (42.0)	
Overall stage			
III	19 (34.5)	12 (24.0)	0.24
IVA	36 (65.5)	38 (76.0)	
IC cycle			
Two	41 (74.5)	34 (68.0)	0.46
Three	14 (25.5)	16 (32.0)	
Concurrent chemotherapy cycle			
None	5 (9.1)	3 (6.0)	0.40
One	5 (9.1)	1 (2.0)	
Two	44 (80.0)	45 (90.0)	
Three	1 (1.8)	1 (2.0)	
Concurrent targeted therapy			
Yes	26 (47.3)	15 (30.0)	0.07
No	29 (52.7)	35 (70.0)	

### Efficacy

After induction therapy, 17 (30.9%), 33 (60.0%) and 5 (9.1%) patients in the GP plus toripalimab group and 6 (12.0%), 41 (82.0%) and 3 (6.0%) patients in the GP alone group achieved complete response (CR), partial response (PR) and stable disease (SD), respectively (p=0.041). There were no patients who had progressive disease (PD) in either group.

**Figure 1:**
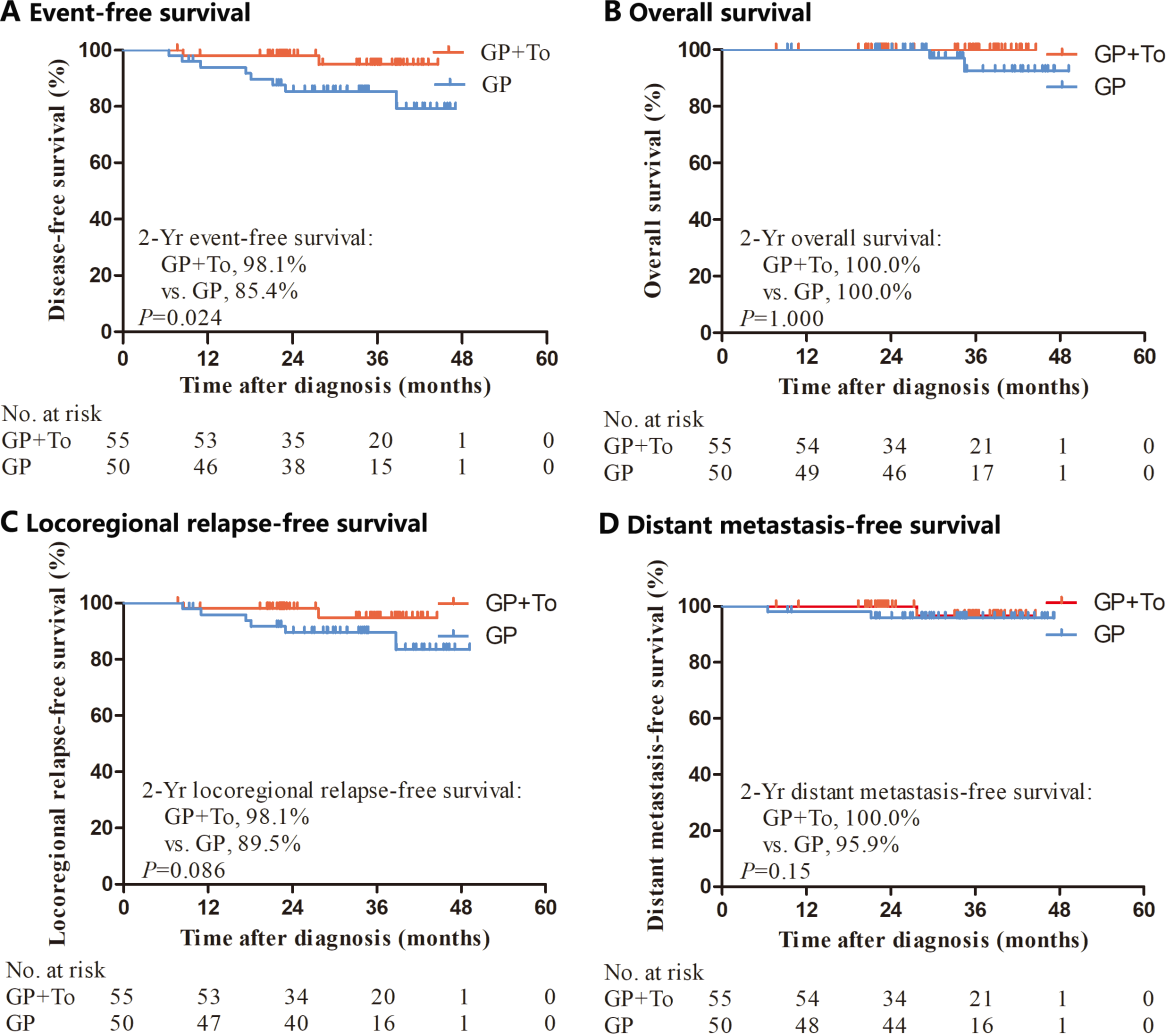
Kaplan–Meier analysis of event-free survival, overall survival, locoregional relapse-free survival and distant metastasis-free survival. GP, Gemcitabine and cisplatin; GP+To, Gemcitabine and cisplatin and toripalimab.

At the last follow-up on June 30, 2024, the median follow-up time was 38.6 months (range, 7.7-53.3). By the cutoff time, 93.3% (98/105) of the patients had been followed for at least 24 months. There were 10 events of disease progression or death (9.5% of patients in the entire population), including 8 of 50 patients (16.0%) in the GP alone group and 2 of 55 (3.6%) in the GP plus toripalimab group. We recorded a total of 2 events of death (1 death 29.5 months after diagnosis and 1 death 34.4 months after diagnosis) in the GP alone group and none in the GP plus toripalimab group. The 2-year EFS was 98.1% in the GP plus toripalimab group and 85.4% in the GP alone group (HR, 0.28; 95% CI, 0.08–0.97; p=0.024) (**Figure 1**). The 2-year OS, LRFS and DMFS rates for the GP plus toripalimab group and the GP alone group were 100.0% and 100.0% (p=1), 98.1% and 89.5% (p=0.086), and 100.0% and 95.9% (p=0.15), respectively (**Figure 1**).

### Adverse events

During induction therapy, acute Grade 3 or 4 adverse events occurred in 20 patients (36.4%) in the GP plus toripalimab group and 21 patients (42.0%) in the GP group (p=0.69). Neutropenia was the most common event (17 patients [30.9%] in the GP plus toripalimab group and 16 patients [32.0%] in the GP group), followed by leukopenia (13 [23.6%] vs. 6 [12.0%]) and nausea (11 [20.0%] vs. 8 [16.0%]) (**Table 2**). During the entire treatment period, 26 patients (47.3%) in the GP plus toripalimab group and 28 (56.0%) in the GP group experienced Grade 3 or 4 adverse events (**Table 3**). Neutropenia was still the most common grade 3 or 4 toxicity (20 patients [36.4%] in the GP plus toripalimab group and 21 [42.0%] in the GP group). Immune-related AEs of grade 3–4 occurred in 3 [5.5%] patients in the toripalimab plus GP arm. The most common immune-related AEs of grade 3–4 were hypothyroidism, cutaneous pruritus, and rash. There were no treatment-related deaths in the two groups.

**Table 2:** Acute toxicity profile during induction therapy.

Toxicity	To+GP (N = 55) (N, %)	GP (N = 50) (N, %)
Major acute event		
G1-2	34 (61.8)	26 (52.0)
G3-4	20 (36.4)	21 (42.0)
Hematological		
Leukopenia		
G1-2	28 (50.9)	34 (68.0)
G3-4	13 (23.6)	6 (12.0)
Neutropenia		
G1-2	23 (41.8)	27 (54.0)
G3-4	17 (30.9)	16 (32.0)
Anemia		
G1-2	30 (54.5)	17 (34.0)
G3-4	0 (0.0)	2 (4.0)
Thrombocytopenia		
G1-2	8 (14.5)	15 (30.0)
G3-4	4 (7.3)	4 (8.0)
Non-hematological		
Liver-function		
G1-2	33 (60.0)	27 (54.0)
G3-4	1 (1.8)	2 (4.0)
Renal-function		
G1-2	7 (12.2)	4 (8.0)
G3-4	0 (0.0)	0 (0.0)
Nausea		
G1-2	20 (36.4)	17 (34.0)
G3-4	11 (20.0)	8 (16.0)
Vomiting		
G1-2	33 (60.0)	32 (64.0)
G3-4	8 (14.5)	6 (12.0)

**Table 3:** Toxicity profile during entire treatment course.

Toxicity	To+GP (N = 55) (N, %)	GP (N = 50) (N, %)
Major acute event		
G1-2	29 (52.7)	22 (44.0)
G3-4	26 (47.3)	28 (56.0)
Hematological		
Leukopenia		
G1-2	34 (61.8)	31 (62.0)
G3-4	18 (32.7)	17(34.0)
Neutropenia		
G1-2	27 (49.1)	26 (52.0)
G3-4	20 (36.4)	21 (42.0)
Anemia		
G1-2	40 (72.7)	40 (80.0)
G3-4	4 (7.3)	7 (14.0)
Thrombocytopenia		
G1-2	14 (25.5)	13 (26.0)
G3-4	10 (18.2)	8 (16.0)
Non-hematological		
Mucositis		
G1-2	32 (58.2)	27 (54.0)
G3-4	10 (18.2)	11 (22.0)
Vomiting		
G1-2	24 (43.6)	19 (38.0)
G3-4	15 (27.3)	12 (24.0)
Nausea		
G1-2	33 (67.3)	35 (70.0)
G3-4	12 (24.5)	10 (20.0)
Dry mouth		
G1-2	47 (85.5)	44 (88.0)
G3-4	2 (3.6)	3 (6.0)
Dermatitis		
G1-2	45 (81.8)	39 (78.0)
G3-4	2 (3.6)	1 (2.0)
Liver-function		
G1-2	32 (58.2)	28 (56.0)
G3-4	1 (1.8)	2 (4.0)
Renal-function		
G1-2	10 (18.2)	9 (18.0)
G3-4	0 (0.0)	0 (0.0)
Major immune-related event		
G1-2	22 (40.0)	N/A
G3-4	3 (5.5)	N/A
Hypothyroidism		
G1-2	13 (23.6)	N/A
G3-4	0	N/A
Cutaneous pruritus		
G1-2	12 (21.8)	N/A
G3-4	1 (1.8)	N/A
Rash		
G1-2	8 (14.5)	N/A
G3-4	2 (3.6)	N/A
Major late event		
G1-2	47 (85.5)	42 (84.0)
G3-4	6 (10.9)	5 (10.0)
Dry mouth		
G1-2	43 (78.2)	39 (78.0)
G3-4	2 (3.6)	2 (4.0)
Deafness or otitis		
G1-2	18 (32.7)	15 (30.0)
G3-4	2 (3.6)	3 (6.0)
Neck tissue damage		
G1-2	11 (20.0)	12 (24.0)
G3-4	0 (0.0)	0 (0.0)

NA, not applicable.

## Discussion

Nasopharyngeal carcinoma is an epithelial malignancy located in the nasopharynx and is closely related to Epstein–Barr virus (EBV) infection. Although NPC is sensitive to radiotherapy and although radiotherapy alone can cure approximately 90% of patients in the early stage, most patients have locoregionally advanced disease at diagnosis.

Radiotherapy combined with chemotherapy is the main treatment strategy for locally advanced nasopharyngeal carcinoma. Induction chemotherapy, which is used before radiotherapy, reduces the tumor burden and is well tolerated by patients. Interestingly, a multicenter, randomized phase III trial revealed that induction chemotherapy with gemcitabine and cisplatin before concurrent chemoradiotherapy significantly improved overall survival in patients with locally advanced nasopharyngeal carcinoma, and there was no increase in late toxicity (9).

Epstein–Barr virus (EBV) is strongly associated with NPC progression and is considered a risk factor for prognosis. EBV−infected epithelial cells often express EBV antigens, which promote the transformation of normal cells into NPC cells (17). Moreover, EBV antigens are the main targets of T cells (18, 19). In addition, tumor tissue is characterized by many immune infiltrates, such as T cells, B cells, dendritic cells (DCs), monocytes and eosinophils (20). In light of these factors, NPC is primarily suitable for immunotherapy.

In the last few years, immune checkpoint inhibitors, as part of immunotherapy, have developed rapidly in the clinical treatment of tumors. Immune checkpoint inhibitors suppress the binding of immunosuppressive signals to the corresponding ligands (e.g., PD−1/PD−L1), ultimately attenuating immunosuppressive regulation, reducing the T-cell suppression state and preventing immune escape. Toripalimab, an immune checkpoint inhibitor, has been shown to have promising efficacy in NPC treatment. The POLARIS−02 trial was a phase II study investigating the efficacy and safety of toripalimab in standard chemotherapy−refractory recurrence or metastasis (R/M) NPC. The results demonstrated that 20.5% of patients achieved an objective response, and the median DoR and OS were 12.8 months and 17.4 months, respectively. Twenty-seven patients (14.2%, 27/190) reported grade 3–5 adverse events (21). Researchers have also evaluated the role of the addition of toripalimab to gemcitabine plus cisplatin chemotherapy as a first-line treatment for RM-NPC. JUPITER−02, a multicenter randomized phase III study enrolling 289 patients with RM-NPC, explored the antitumor activity and toxicity of toripalimab or placebo plus GP as first−line care for patients. The data revealed that the PFS in the toripalimab group was markedly prolonged compared with that in the placebo group (11.7 versus 8.0 months, p=0.0003). Compared with the placebo group, the toripalimab group had a higher objective response rate (77.4% vs. 66.4%, P = 0.0335). There was no significant difference in adverse reactions ≥ grade 3 between the two groups (89.0% vs. 89.5%) (14). In accordance with the JUPITER−02 trial, toripalimab alone or in combination with chemotherapy was approved as the first−line treatment for patients with R/M NPC in China.

More and more studies reported that adding immune checkpoint inhibitors into the primary treatment for LANPC increased the progression-free survival, with manageable toxicity. However, the type, dosage, and timing of integration (induction phase, concurrent phase, and adjuvant phase) of immune checkpoint inhibitors into standard primary treatment of LANPC varies among these studies (22–24). Interestingly, a randomised, double-blind, phase 2 trial demonstrated that a so-called sandwich approach involving toripalimab (in the neoadjuvant and adjuvant phases) combined with concurrent chemoradiotherapy could be a highly promising therapy for the treatment of LANPC (25). During the induction period, chemotherapy was not administered and only immunotherapy was employed in the trial (25). In addition, a recent study has shown toripalimab combination therapy without concurrent cisplatin was a feasible therapy with high efficacy in failure-free survival and low toxicity in LANPC (26). Several studies have also shown induction immunochemotherapy combined with concurrent chemoradiotherapy has promising antitumor activity with a manageable safety profile in patients with LANPC (27–30). However, the role of gemcitabine and cisplatin induction chemotherapy plus toripalimab in LANPC was unclear.

In this retrospective study, we explored the efficacy and toxicity of the addition of toripalimab to GP induction chemotherapy in locoregionally advanced nasopharyngeal carcinoma patients. The majority of patients with poor prognostic factors had N2 or N3 disease or T3 or T4 nasopharyngeal lesions. Our data revealed that the complete response rate after induction therapy was 30.9% (17/55) in the toripalimab plus GP group, which was obviously higher than that (12.0%, 6/50) in the GP group. The 2-year event-free survival rate was 98.1% in the toripalimab plus GP group and 85.4% in the GP group, which was significant (p=0.024). Grade 3–4 acute AEs during induction treatment occurred in 20 (36.4%) patients in the toripalimab plus GP group versus 21 (42.0%) in the GP group. The most common grade 3–4 acute AEs were neutropenia (17 [30.9%] vs. 16 [32.0%]) and leukopenia (13 [23.6%] vs. 6 [12.0%]) between the toripalimab group and the GP alone group. No patient died in either group 2 years after treatment. In addition, There were 3 [5.5%] patients having immune-related AEs of grade 3–4 in the toripalimab group. Liu X et al. reported that grade 3–4 immune-related AEs occurred in 10% (20/200) of patients after the addition of sintilimab (a PD-1 inhibitor) to standard therapy (GP induction chemotherapy followed by concurrent cisplatin radiotherapy) for 12 cycles (3 induction, 3 concurrent, and 6 adjuvant cycles) in high-risk LANPC patients (31). There may be two reasons for the difference in immune-related AEs between the two studies. First, the internal structures of toripalimab and sintilimab are different. Second, the total dose of toripalimab (240 mg once every 3 weeks for 2 or 3 cycles) used in the induction phase was less than that of sintilimab (200 mg once every 3 weeks for 12 cycles) used in the whole treatment phase. In our study, we excluded patients who received adjuvant immunotherapy because we wanted only to explore the role of induction immunotherapy in LANPC, and the number of patients receiving adjuvant immunotherapy was very small due to COVID-19.

However, this study had several limitations. First, this was a retrospective study, and the sample size was small, resulting in potential biases. Second, the follow-up duration may have been insufficient. Third, for a period of time, due to the EBV-DNA testing method, the positive rate of EBV-DNA was low. After the improvement of testing method, the positive rate became more accurate. The testing methods were inconsistent before and after, so this data is not included in this study.

In summary, the addition of toripalimab to GP induction chemotherapy significantly improves the EFS of LANPC patients in the era of IMRT, and toxicity is tolerable. Nevertheless, these findings need to be validated in prospective clinical trials.

## Data Availability

The raw data supporting the conclusions of this article will be made available by the authors, without undue reservation.
